# A self-guided Internet-based intervention for individuals with gambling problems: study protocol for a randomized controlled trial

**DOI:** 10.1186/s13063-019-3176-z

**Published:** 2019-01-23

**Authors:** Lara Bücker, Stefan Westermann, Simone Kühn, Steffen Moritz

**Affiliations:** 1 Department of Psychiatry and Psychotherapy, University Medical Center Hamburg-Eppendorf, Martinistrasse 52, 20246 Hamburg, Germany; 2 Department of Psychology, UC Berkeley, 2121 Berkeley Way West, 94720 Berkeley, CA USA

**Keywords:** Internet-based self-help, CBT, Problematic gambling, Pathological gambling, Online intervention

## Abstract

**Background:**

Only a small fraction of individuals with pathological or problematic gambling seek professional help despite available evidence-based treatments such as cognitive behavioral therapy (CBT). Anonymous Internet-based interventions may help to overcome treatment barriers. Results of a pilot study using an Internet-based intervention for depression in a sample of individuals with problematic or pathological gambling behavior show that both depressive and gambling-related symptomatology can be reduced with a generic depression program compared with a wait-list control group. Based on encouraging results of the pilot study, we developed a low-threshold, anonymous and cost-free online self-help program (“Restart”) to test whether a program tailored to the needs of gamblers yields better results compared to the effects of the intervention evaluated in the pilot study. The online self-help program is based on CBT, targeting emotional problems and gambling-related symptoms and is accompanied by a smartphone application to sustain treatment benefits.

**Method:**

A randomized controlled trial with two conditions (intervention group and wait-list control group), two assessment times (reassessment after 8 weeks) and a total of 136 participants is planned. The primary outcome will be change in pathological gambling measured with the Pathological Gambling Adaptation of Yale-Brown Obsessive-Compulsive Scale from pre to post intervention. The change in depressive symptoms (assessed with the Patient Health Questionnaire - 9 depression module) and gambling-related dysfunctional thoughts (assessed with the Gambling Attitudes and Beliefs Survey) will represent secondary outcomes. The intervention includes modules on debt management, impulse control, gambling-specific cognitive biases, self-esteem, social competence, sleep hygiene, mindfulness and positive activities.

**Discussion:**

This study is one of the first investigations of Internet-based self-help programs in a sample of problematic gamblers. Self-guided Internet-based interventions represent a promising possibility to narrow the existing treatment gap while saving expensive and scarce resources (e.g., psychotherapists). The expected findings will add substantial knowledge in the development of effective Internet-based treatments for individuals with gambling problems. The empirical and clinical implications (e.g., broader use and promotion of such interventions in the future) and the limitations of the study will be discussed.

**Trial registration:**

ClinicalTrials.gov, NCT03372226. Registered on 13 December 2017.

**Electronic supplementary material:**

The online version of this article (10.1186/s13063-019-3176-z) contains supplementary material, which is available to authorized users.

## Background

Many people dream of a big win and according to a survey of the German Federal Center for Health Information [1] 77.6% of the 16–70 year-old German population have had a gambling experience at least once in their life. For some, this dream turns out terrible and what once used to be an entertaining hobby becomes a profound problem. Slot-machine gambling has been described as the most harmful and addictive gambling form, which is also reflected by the fact that most of those seeking help report this form of gambling [2, 3].

Pathological gambling is characterized by persistent and recurrent problematic gambling behavior leading to clinically significant impairment or distress [4]. The individuals affected experience a loss of control over their gambling activity and have persistent thoughts of past gambling experiences, upcoming ventures and every possible way to obtain money. More and more money is invested in order to experience the desired excitement or to chase losses. The urge to play - despite obvious negative consequences - is often driven by a variety of gambling-specific cognitive biases such as illusion of control (perceiving control over an uncontrollable event), gambler’s fallacy (overestimating the probability of predicting an event), chasing (the belief that after a significant loss, money can only be recovered through continued gambling), superstitious beliefs (e.g., assigning lucky attributes to objects, behaviors, etc.), misattributions (external and internal) and magical thinking (the belief that behaviors that are not causally related to the outcome can influence the probability of winning) (for an overview of gambling-specific cognitive biases see [2]). There is evidence that the presence of those cognitive biases is involved in the maintenance of problematical and pathological gambling behavior [5, 6]. Therefore, the biases should be more thoroughly addressed and focused on in the treatment of gambling disorders, for example by using strategies referred to as cognitive restructuring (CR) that help clients understand that their thoughts are irrational [7].

Furthermore, depressive symptoms are associated with problematic and pathological gambling. Although there is consensus that depressive symptoms are often observed in pathological gamblers, the exact causal relationship between both is disputed. Several models for categorizing gamblers into types have been proposed [8–10]. Each of these models describes at least two types of gamblers - those who gamble to reduce preexisting negative emotions and those who develop depressive symptoms in response to gambling-related problems such as financial difficulties [9]. As one to two thirds of the individuals affected appear to have comorbid depressive symptoms, they should definitely be addressed in therapy [11].

Despite numerous negative consequences and suffering, only a small fraction of those affected receive professional treatment [12] and attrition rates are high for those who start therapy [13, 14]. There is evidence on the effectiveness of psychological treatments for pathological gambling [15], with cognitive behavioral therapy appearing to be the most effective [16, 17]. However, conventional “face-to-face” therapies do not seem suitable to fill the large treatment gap [12], possibly because the treatment barriers to those forms of therapy are too high, so alternative possibilities must be considered.

Internet-based interventions have been investigated and evaluated in recent years in many studies of different mental disorders, with promising outcomes [18–20]. Benefits of Internet-based interventions include its anonymity and privacy, low costs, flexibility and accessibility [21]. This enables them to overcome treatment barriers of conventional therapies such as fear of stigma, shame and geographical or time limitations. Internet-based intervention may be guided or self-guided (unguided). Guided interventions are therapist-supported (e.g., by e-mail or telephone support), whereas self-guided interventions do not provide individual support by a trained person or psychotherapist. Studies on guided Internet-based interventions consistently report greater effect sizes than unguided Internet-based interventions [22]. Moreover, a recent meta-analysis that compared guided Internet-based interventions with face-to-face cognitive behavioral therapy (CBT) found comparable effect sizes [18]. However, smaller effect sizes in studies of self-guided Internet-based interventions nevertheless demonstrate their effectiveness [23]. Although they are less effective than guided Internet-based interventions, they have some advantages as they are more cost-effective, resource-efficient and flexible. As adherence is a problem in self-guided Internet-based interventions, it is necessary to provide strategies that enhance adherence [24]. According to a review [25], it seems that sending e-mail reminders and presenting information in textual format (instead of non-textual such as audio/video format) contributes to treatment success.

The first attempts to investigate Internet-based interventions for pathological gamblers have been made in recent years [26]. One study tested the effectiveness of an Internet-based self-help program with weekly telephone support and e-mail contacts in a sample of pathological gamblers with no comorbid severe depressive symptoms and found positive changes in pathological gambling, anxiety, depression and quality of life [27]. A randomized controlled trial (RCT) tested the efficacy of three modalities of Internet-based psychotherapy (three conditions with or without guidance and a control condition), among problem gamblers participating in online poker [28] but did not find a significant difference between the groups in treatment outcome. Guidance here means human support in the form of weekly e-mails with personalized CBT. In all groups there were high attrition rates (83%); interestingly, the attrition rate was higher in the group receiving guidance (95.3% after 6 weeks, 97.3% after 12 weeks) compared to the other experimental groups, tentatively indicating that guidance has adverse effects in Internet-based interventions for problem gamblers. In a pilot study, we investigated whether a self-help online program for depressive symptoms “Deprexis” has positive effects on depressive and gambling-related symptoms [29]. This intervention led to a significant reduction in depressive symptoms and gambling-related symptoms, compared to a control group. However, the results gave rise to the hypothesis that treatment effects are likely augmented if the program is tailored to the specific needs of problem gamblers.

The efficacy of Internet-based interventions has been shown in many trials in other psychiatric disorders and the first results from these studies of pathological gambling are promising. However, studies in this particular field, especially randomized controlled studies, are rare. Building upon the results of the pilot study, our study group developed an Internet-based intervention, called “Restart”, that addresses gambling-related and emotional problems. The Internet-based intervention is accompanied by a smartphone application (app) that sends daily reminders, with small exercises that can be easily integrated into everyday life, to increase therapeutic success. A review has indicated the efficiency of smartphone-supported self-help [30].

The aim of the study is to evaluate the efficacy and acceptance of the Internet-based intervention, Restart, which is aimed at treating gambling-related and emotional problems. We expect that the Internet-based intervention will lead to a significant reduction in pathological gambling (primary treatment target) as measured by the Pathological Gambling Adaptation of Yale-Brown Obsessive Compulsive Scale (PG-YBOCS), gambling-specific cognitive distortions (measured by the Gambling Attitudes and Beliefs Survey (GABS)) and depressive symptoms (measured by the Patient Health Questionnaire - 9 (PHQ-9) depression module) when compared with a control group that receives no treatment.

## Methods

### Study design

**Fig. 1:**
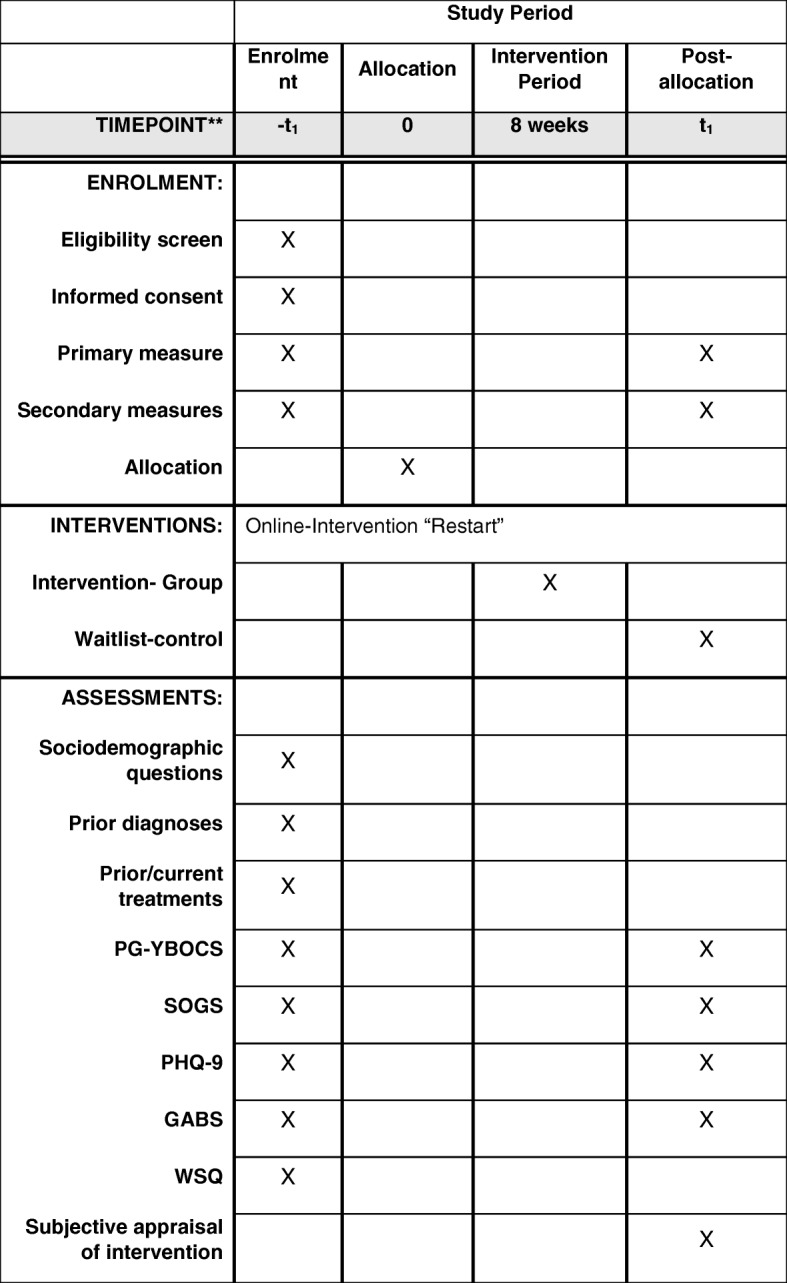
Standard protocol items: recommendation for interventional trials (SPIRIT) timeline. PG-YBOCS, Pathological Gambling Adaption of Yale-Brown Obsessive-Compulsive Scale; SOGS, South Oaks Gambling Screen; PHQ-9, Patient Health Questionnaire-9; GABS, Gambling Attitudes and Beliefs Survey; WSQ, Web Screening Questionnaire

The self-help program is evaluated in the framework of a RCT with parallel assignment to two conditions. The intervention group receives Restart over a period of 8 weeks and will be compared with a wait-list control group, which receives full access to the program following the post-intervention assessment. There are two assessment points (baseline and post intervention), where data are obtained by an Internet survey using Unipark®. At baseline, sociodemographic and psychopathological data are assessed; at the post-intervention assessment, psychopathological data and subjective evaluations of the Internet-based intervention (only for the Restart intervention group) are assessed. Within the online baseline assessment, participants are screened for inclusion and exclusion criteria before randomization. All participants will be invited to participate in the post-intervention assessment 8 weeks after the baseline assessment. As an incentive, the completion of the follow-up assessment will be rewarded with a 20 Euro Amazon voucher and all participants will receive access to another Internet-based training intervention to reduce the problematic gaming behavior (retraining). See Fig. 1 for a schedule of enrollment, interventions and assessments (SPIRIT).

### Sample size

The sample size was calculated using G*Power [31], which indicated an entire sample size of 90 to detect a medium effect of *d* = 0.5, with α = 0.05 and power of 0.80 for analysis of covariance (ANCOVA). Based on previous studies of adherence in Internet-based interventions [32, 33] and among pathological gamblers [34], we expect a dropout rate of 50%. Taking this into account, the total sample size should be 136 with 68 participants in each condition.

### Recruitment

Participants will be recruited via a Google AdWords campaign. Depending on the words a person enters into the Google search engine, certain pages are suggested to them and most people look at the top results of the Google search. Here it is possible to place an advertisement for a fee per click, which is then displayed directly at the top. In our study, such an advertisement will direct the user to a website with information about the study and a further link to the online baseline assessment. The advertisement for Restart is displayed by entering relevant keywords such as “problematic + gambling + help”. The Google AdWords campaign will be launched in Germany, (German-speaking part of) Switzerland and Austria.

### Eligibility criteria

The participants have to fulfill the following criteria to be included in the study:

1. Age between 18 and 75 years
2. Agreement to participate via electronic informed consent
3. Internet access
4. Sufficient command of the German language
5. Willingness to participate in two online surveys
6. Willingness to participate in the self-help training over a period of 8 weeks
7. Willingness to provide an (anonymous) e-mail address (we provide information about how to set up an anonymous e-mail address without including their real name)
8. Presence of psychological strain, subjective feelings about having a problem with gambling and desire for treatment (participants with a South Oaks Gambling Screen (SOGS) sum score < 1 will be excluded from the analyses)

Individuals are not included in the study if they have a lifetime diagnosis of a schizophrenia spectrum or bipolar disorder or if they are acutely suicidal (assessed by one item on suicidality of the PHQ-9). Suicidal individuals are provided with help facilities and telephone numbers to contact in acute cases.

### Randomization

Participants will be randomly allocated to one of the two conditions (intervention group or wait-list control group). The randomization plan will be created from an independent researcher by using block randomization with four participants per block to assure that group sizes are equal. There is no need for concealment of allocation, as allocation here differs from the standard procedure in which study members actively approach participants and where there is previous contact between the researcher (or person who is performing the allocation) and the participants. In this study, participants click on the Google advertisement and if they are interested in participating they start with the baseline online assessment. After completion of the baseline assessment, the researcher obtains the information by e-mail that a new participant has completed the baseline assessment. He then allocates the participants to the two conditions according to the randomization plan. This is performed chronologically, i.e. based on the time and date of completion of the baseline assessment, to prevent any arbitrary allocation of participants. The person (first author) who is allocating the participants thus has no influence on the randomization and enrollment process.

### Intervention

The Internet-based intervention, Restart, consists of 11 modules that are based on CBT principles but also includes elements of its third wave, including mindfulness-based and metacognitive techniques. Comorbid emotional and mood problems are also addressed in the program, as they often accompany gambling problems and are assumed to be involved in the onset and maintenance of problematic and pathological gambling [9, 35]. There are more generic modules, e.g., focusing on sleep hygiene, positive activities, social competence and self-worth, however, all modules address pathological gambling with case examples. Furthermore, there are modules on the regulation of debts, handling gambling impulse, adapting gambling-specific thought distortions and relapse prevention, which are more specific to pathological gambling (see Table 1 for an overview). The modules all have an interactive format with exercises and worksheets. The program is text-based with a number of audio files (e.g., mindfulness-based relaxation and breathing exercises) and videos with psychoeducational content (e.g., about a model of the development of sleep disturbances) to make the intervention more appealing. Participants can freely choose in which order they want to work on the modules. We recommend participants to work on one or two modules per week. The duration of each module varies from 30 to 60 min. The participants have the opportunity to communicate with a personal moderator (optional). The moderator helps in case the participant has questions about the program or problems with exercises. Messages are answered within three workdays. If the participant does not use the program, the moderator reminds the participant to work with the program by sending messages including a program overview to enhance adherence. Furthermore, a smartphone app can be used in combination with the desktop Internet-based intervention. The app provides a total of 50 small exercises that refer to content of Restart but is otherwise independent of the portal. The app sends daily reminders via push messages at freely definable times, so that users remember to take time for their mental health in everyday life.

**Table 1:** “Restart” modules

Module Title	Description
Introduction	Introduction to features of the program; demonstration of the interaction between thoughts, emotions and behavior
ABC	Introduction to a psychological method to understand how certain feelings and behaviors arise
Handling of gambling impulse	Detection of triggers and strategies to deal with the urge to gamble
Money	Development of monthly/daily lists of debts, expenses and income; Learn strategies to resist the temptation to spend money thoughtlessly
Positive activities	Gives advises on how to integrate positive activities into daily routine; goal formulation; effective planning of activities and long-term goals
Self-worth	Detection of own strengths and valuing self-perception
Social competence	Demonstration of strategies of expedient, cooperative and firm communication
Mindfulness Sleep hygiene	Presentation of mindfulness-based relaxation and attention exercises (e.g., relaxation and breathing exercises, body scan, yoga elements) Explains the importance of sleep quality and gives advice on improving sleep hygiene.
Modifying thoughts	Gives advises on how to adapt depressive and gambling-specific dysfunctional thoughts (e.g., negative filter, gambler’s fallacy, illusion of control) into more realistic/balanced thoughts; metacognitive elements
Relapse prevention	Identification of warning signals for relapse and find strategies to deal with those

### Measures

The primary outcome measure is the post-assessment reduction in gambling-related symptoms, for example, thoughts on gambling and gambling behavior (as assessed by the PG-YBOCS [36]). Secondary outcomes include the reduction in depressive symptoms and gambling-related dysfunctional thoughts (assessed by the PHQ-9 and GABS).

### Primary measures

#### Pathological Gambling Adaptation of Yale-Brown Obsessive Compulsive Scale (PG-YBOCS)

The PG-YBOCS [36] assesses the severity of gambling symptoms. The questionnaire includes two subscales: urges and thoughts associated with gambling and actual gambling behavior. The sum score of each subscale ranges from 0 to 20. The total score can be interpreted as follows: 0–7 sub-clinical, 8–15 mild, 16–23 moderate, 24–31 severe and 32–40 extreme gambling symptoms. The time frame is the past week.

### Secondary measures

#### Patient Health Questionnaire - 9 depression module (PHQ-9)

The PHQ-9 [37, 38] is a self-rating questionnaire to measure depressive symptom severity. The PHQ-9 has high internal consistency (Cronbach’s α = 0.86–0.89; [38]). Its sum score ranges from 0 to 27, with scores from 0 to 4 indicating minimal depression, 5–9 mild depression, 10–14 moderate depression and 15–27 severe depression. The time frame is the past week.

#### Gambling Attitudes and Beliefs Survey (GABS)

The GABS [39] is a self-report questionnaire that explores gambling-related dysfunctional beliefs, such as illusion of control and superstitious thinking. We will use a 15-item short version of the GABS, with all items that were examined when using item response theory [40]. Internal consistency of the whole scale is reported as good (Cronbach’s α > 0.9) [41]. Responses must be given on a 4-point Likert scale (from “strongly agree” to “strongly disagree”). The questionnaire refers to the current state of assessment time.

#### South Oaks Gambling Screen (SOGS)

The SOGS [42] is a 20-item self-report measure to assess engagement in gambling activities and problems related to gambling. The questionnaire is the most frequently used instrument to measure gambling problems internationally and has moderate internal consistency (Cronbach’s α = 0.69) and good convergent validity, as it is strongly correlated with both the *Diagnostic and Statistical Manual of Mental Disorders* (DSM)-IV and DSM-5 criteria for pathological gambling (*r* = .66) [43]. We use the SOGS as a screening instrument for pathological gambling. Sum scores of 0–2 designate non-problematic gambling, scores of 3–4 designate at-risk gambling and scores of 5–20 designate pathological gambling.

#### Web Screening Questionnaire (WSQ)

The WSQ [44] is a brief online self-report screening instrument that assesses the following common mental disorders: depressive disorders, alcohol abuse/dependence, general anxiety disorder, posttraumatic stress disorder, social phobia, panic disorder, agoraphobia, specific phobia, obsessive compulsive disorder and suicide risk. The screening instrument has values for sensitivity ranging between 0.72 and 1.00 and for specificity between 0.44 and 0.77. Participants will only be screened at baseline.

#### Subjective appraisal of the program

The subjective evaluation of the program is assessed by 15 specially created questions on the quality, utility and applicability of Restart (see Additional file 1) and by the German version [45] of the Client Satisfactory Questionnaire (CSQ-8) [46].

### Statistical analyses

Both the intention-to-treat (ITT) and per-protocol samples will be analyzed using linear mixed-model repeated measures ANCOVA with pre-to-post intervention time as the within-group factor, condition as the between-group factor and baseline scores as the covariate. Baseline differences between the two groups will be assessed using the independent samples *t* test for continuous variables and the chi-square test for categorical variables. Missing values for ITT analyses will be imputed using two different methods: (1) maximum likelihood estimation and (2) multiple imputation. In addition, we will conduct an exploratory moderation analysis to see which factors potentially have a moderating influence on symptomatic improvement using the SPSS macro PROCESS (developed by A. Hayes).

### Ethical aspects and data safety

The study was approved by the German Society for Psychology (DGPs) (ID: SM 092017_amd_012014_2b) and will be conducted in accordance with the Declaration of Helsinki. At no point is personal information such as name, telephone number or address requested. The participants have only to provide an anonymous e-mail address (in which they do not provide any names). Instructions for creating an anonymous e-mail address are given. All data collected, except for the e-mail addresses (which are stored non-electronically), is stored on password-protected computers that are known only to the respective computer user. The anonymized data will be archived for 10 years after completion of the study. The data already collected will be deleted in the case of revocation of the declaration of consent. All email addresses will be obliterated after completion of the data collection. The network security of Restart is achieved by using secure sockets layer (SSL) encryption. Apart from the people directly involved in the project, no third parties will have access to the final trial dataset. The duration that the individual participant will be logged into the program can be checked by us via the logfiles of the program.

## Discussion

The Internet-based intervention, Restart, is one of the first online programs that addresses gambling-related problems. Evidence suggests that Internet-based interventions can be effective in reducing pathological gambling [27, 28]. Our study aims at reaching those who do not seek conventional face-to-face therapy, thereby narrowing the existing treatment gap. Restart has several advantages over conventional face-to-face therapies, such as low costs, anonymity and high accessibility and flexibility. The Internet-based intervention is based on CBT, which is currently reported to be the most effective therapy in the treatment of pathological gambling [15–17], and its third wave. In addition, Restart addresses not only gambling-related problems but also mood problems and helps the user to strengthen self-worth, elaborate new strategies for positive social interactions and to adapt dysfunctional thoughts to more realistic and positive thoughts. Here, depression-specific and gambling-specific dysfunctional thoughts are addressed. We expect that the intervention significantly reduces gambling-related symptoms, gambling-specific dysfunctional thoughts and depressive symptoms when compared with a control group. We wanted to offer completely anonymous study participation, so it is not possible to have third-party ratings and to confirm diagnoses by means of structured interviews. This would have also raised the threshold for participation. Last, since we expect an even higher drop-out rate at later follow-up assessments, we have decided to include only two assessment time points and therefore cannot draw any conclusions on the long-term effects. Nevertheless, this study promises to add substantial knowledge to our understanding of Internet-based interventions for pathological gambling. Furthermore, future implications and possibilities of the Internet-based interventions will be discussed. For example, it is conceivable that Restart could reach those who are not looking for classical face-to-face therapy, who do not have access to psychotherapy (e.g., due to geographic distances) or it could be integrated into stepped-care models. Restart could represent a first step of professional help, possibly for individuals who have slight symptoms or for those who are still waiting to receive therapy.

### Trial status

The trial started on 20 January 2018. At the time of submission, the recruitment was ongoing and will presumably be completed in February 2019. Any future changes to the study protocol will be recorded in a separate amendment. Throughout the article we follow the SPIRIT guidelines (see Additional file 2 for the checklist). 

## Additional file

Additional file 1: **Table S1.** Subjective evaluation of the program. (DOC 37 kb)

Additional file 2: **Table S2.** SPIRIT 2013 Checklist. (DOC 101 kb)

## Abbreviations

ANCOVA: Analysis of covariance
App: Application
CBT: Cognitive behavioral therapy
DSM-5: *Diagnostic and statistical manual of mental disorders (5th Edition)*
GABS: Gambling Attitudes and Beliefs Survey
ICD-10: International Classification of Diseases and Related Health Problems (10th revision)
MBCT: Mindfulness-based cognitive therapy
PG-YBOCS: Pathological Gambling Adaption of Yale-Brown Obsessive-Compulsive Scale
PHQ-9: Patient Health Questionnaire-9
RCT: Randomized controlled trial
SOGS: South Oaks Gambling Screen
WSQ: Web Screening Questionnaire
