# Supplementary material for: A self-guided Internet-based intervention for individuals with gambling problems: study protocol for a randomized controlled trial
Source: Trials. 2019 Jan 23;20:74. doi: 10.1186/s13063-019-3176-z (PMC6343284; doi:10.1186/s13063-019-3176-z)
Supplement: Supplementary file 1 — Table S1. Subjective evaluation of the program. (DOC 37 kb) [file 13063_2019_3176_MOESM1_ESM.doc]

| **1. I think the program is suitable for self-application.** | | - **Yes** - **No** |
| --- | --- | --- |
| **2. My gambling problem reduced by using the program.** | | - **Yes** - **No** |
| **3. I think the instructions were written comprehensibly.** | | - **Yes** - **No** |
| **4. I think the program was useful.** | | - **Yes** - **No** |
| **5. I was able to use the program regularly over the past weeks.** | | - **Yes** - **No** |
| **6. I had to push myself to use the program.** | | - **Yes** - **No** |
| **7. I consider the program to be a useful adjunct to psychotherapy.** | | - **Yes** - **No** |
| **8. The program is not relevant for my gambling-related symptoms.** | | - **Yes** - **No** |
| **9. How many days in the past 8 weeks have you used the program?** | | **days** |
| **10. I particularly liked the following about the program:** | | |
| **11. I didn't like the following or it should be improved:** | | |
| **12.** **Do you plan to continue using the program in the future?** | | - **Yes** - **No** |
| **13.** **Did the program meet your expectations?** | - **Yes** - **No, I would have liked the following:** | |
| **14.** **Has the use of it changed your willingness and motivation to take other treatment measures?** | **Psychotherapy:**   - **reduced** - **remained the same** - **increased**   **Self-help groups:**   - **reduced** - **remained the same** - **increased**   **Self-help programs on the Internet:**   - **reduced** - **remained the same** - **increased** | |
